# Co-presentation of unilateral femoral and bilateral sciatic nerve variants in one cadaver: A case report with clinical implications

**DOI:** 10.1186/2045-709X-20-34

**Published:** 2012-10-29

**Authors:** Patrick J Battaglia, Frank Scali, Dennis E Enix

**Affiliations:** 1 Division of Research, Logan College of Chiropractic, 1851 Schoettler Rd, Chesterfield, MO, 60317, USA; 2 Independent Researcher, 1272 Dutch Broadway, Valley Stream, NY, 11580, USA

**Keywords:** Sciatic nerve, Femoral nerve, Disc, Variant

## Abstract

**Objective:**

To present a group of anatomical findings that may have clinical significance.

**Design:**

This study is an anatomical case report of combined lumbo-pelvic peripheral nerve and muscular variants.

**Setting:**

University anatomy laboratory.

**Participants:**

One cadaveric specimen.

**Methods:**

During routine cadaveric dissection for a graduate teaching program, unilateral femoral and bilateral sciatic nerve variants were observed in relation to the iliacus and piriformis muscle, respectively. Further dissection of both the femoral nerve and accessory slip of iliacus muscle was performed to fully expose their anatomy.

**Results:**

Piercing of the femoral nerve by an accessory iliacus muscle combined with wide variations in sciatic nerve and piriformis muscle presentations may have clinical significance.

**Conclusions:**

Combined femoral and sciatic nerve variants should be considered when treatment for a lumbar disc herniation is refractory to care despite positive orthopedic testing.

## Background

The recurrence of leg pain from lumbar disc herniations is a common post treatment clinical finding. Certain muscular and peripheral nerve variants may represent an unrecognized etiology in these cases and may affect the outcome of specific treatments. Recognition of these variations in normal anatomy may be useful to the clinician when treating the patient with refractory leg pain. The femoral nerve, derived from the second to fourth lumbar dorsal divisions, is one of the terminal branches of the lumbar plexus [1]. Multiple studies have reported variant slips of the psoas and iliacus muscles which may split the femoral nerve causing a potential risk for nerve entrapment [2-9]. In a large study of 121 cadavers, Vazquez et al. reported variations of iliacus and psoas muscles piercing the femoral nerve, piercing of the femoral nerve by a muscular slip, or a muscular slip/sheet covering the femoral nerve as it lay on the iliacus in 19 specimens (7.9%) [3]. Several entities exist which may cause femoral neuropathy [10], however, owing to the lack of anatomic protection, entrapment of the nerve is most likely to occur immediately distal to the inguinal ligament [10]. No clinical finding is pathognomonic for femoral neuropathy, as similar findings of absent or diminished patellar reflex, quadriceps weakness or wasting, weakness in hip flexion and adduction as well as sensory symptoms such as pain in the iliac fossa, inguinal region, anterior thigh and medial calf may also indicate radiculopathy, plexopathy, or combined lesions of the femoral and obturator nerves [11].

The sciatic nerve, formed from the ventral rami of the fourth lumbar to third sacral spinal nerves, leaves the pelvis passing both anterior and inferior to the piriformis or sometimes through the muscle [1]. A 2010 literature review reported that the prevalence of piriformis and sciatic nerve variants in a large sample size of 6,062 cadaveric specimens was 16.9% [12]. The relationship of the piriformis and sciatic nerve causing piriformis syndrome remains a controversial condition. Most commonly, sciatic neuropathy is iatrogenic, occurring after total hip arthroplasty [13]. Yuen, commenting on numerous studies, estimated the frequency of sciatica neuropathy after total hip arthoplasty to be between 0.7% and 3.7% [13]. Numerous other etiologies for sciatic neuropathy and sciatica pain exist [13,14]. Clinical exam findings of observed foot drop, and motor, sensory and reflex deficits in the sciatic nerve distribution are non-specific for sciatic neuropathy and mimic lesions to the lumbosacral nerve roots or plexus [13].

Piercing of the femoral nerve by an accessory iliacus muscle in combination with bilateral variations in both sciatic nerve and piriformis muscle anatomy exemplifies the wide variability that exists within the lumbar and lumbosacral plexus. The clinical implications of these combined variants are discussed.

## Case presentation

During routine cadaveric dissection, bilateral sciatic and unilateral femoral nerve variants were detected. The course and muscular relationships of both sciatic nerve variants were studied. The femoral nerve variant was further dissected and was examined to determine its nerve root contributions and its branching pattern. Also, the accessory muscular slip of the iliacus muscle that was piercing the femoral nerve was dissected to determine both its origin and insertion points.

**Figure 1:**
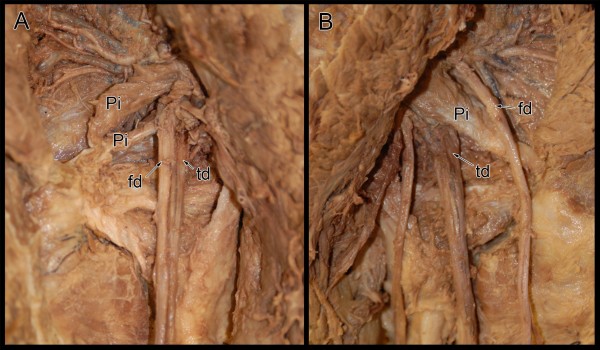
**Photograph reveals bilateral sciatic nerve variants on a single cadaveric specimen.** The left side (**A**) revealed that sciatic nerve divided proximal to the piriformis muscle. The left common fibular division (fd) divided the piriformis (Pi) into two distinct bellies while the left tibial division (td) passed inferior and deep to the most caudal border of the piriformis muscle. On the right side (**B**), the sciatic nerve was split into the right common fibular (fd) and tibial (td) divisions proximal to the piriformis muscle (Pi). The right common fibular division passed superficial while the tibial division passed deep to the piriformis muscle.

On the right side, the sciatic nerve was split into the common fibular and tibial divisions proximal to the piriformis muscle, with the common fibular division passing above and superficial to the piriformis muscle and the tibial division passing inferior and deep to the muscle. On the left side, the sciatic nerve was also divided proximal to the piriformis muscle. However, the piriformis muscle was pierced and subdivided into two discrete bellies by the common fibular division, while the tibial division passed inferior and deep to the most caudal border of the piriformis muscle [Figure 1]. The right piriformis was one discrete muscle. On both sides the remaining course and distribution of the tibial and common fibular nerves was considered normal.

**Figure 2:**
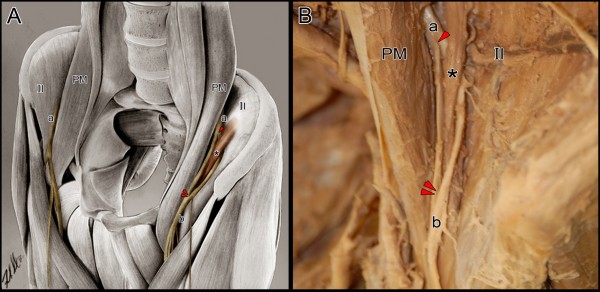
**Illustration (A) and photograph (B) reveals unilateral (left side) femoral nerve variant.** The femoral nerve (**a**) emerged bilaterally lateral and deep to the psoas major muscle (PM). While the right femoral nerve maintained its usual path, the left femoral nerve was pierced (arrowhead) and divided into two separate divisions by an accessory slip of the iliacus muscle (*). These two separate divisions of the femoral nerve converged (double arrowheads) into a single division (**b**) and divided into its usual branches. Also labeled bilaterally is the iliacus muscle (Il). (Original anatomical illustration by Frank Scali, DC).

In the left iliac fossa, the femoral nerve emerged both lateral and deep to the psoas major muscle between the psoas major and iliacus muscles covered in iliac fascia. It was then pierced and divided into two separate divisions by an accessory slip of the iliacus muscle. Just proximal to the inguinal ligament, these two separate divisions rejoined and the femoral nerve passed as one under the inguinal ligament and then divided into its usual anterior and posterior branches [Figure 2]. The accessory slip of iliacus muscle was then dissected proximally up to its origin on the inferior aspect of the iliac crest. It was detached from its origin confirming it had no attachment to the iliolumbar ligament. The muscular slip was then followed distally until it blended into other iliacus and psoas major fibers to incorporate into the iliopsoas muscle which inserted on the lesser trochanter of the femur. The psoas minor and major muscles were then reflected to expose the lumbar plexus. The femoral nerve was found to be formed from the posterior division of the L2, L3 and L4 ventral nerve roots and was fully formed prior to being pierced by the accessory slip of the iliacus muscle. No other lumbar plexus variations were detected. The femoral nerve on the right side of the specimen followed a routine course.

## Conclusions

The sciatic and femoral nerves represent the two largest peripheral collections of lumbar and sacral nerve roots [1]. There have been other cadaveric reports of variance in sciatic and femoral nerve as well as piriformis and iliopsoas complex muscle anatomy similar to what is described in this case [2-9,12,15]. However, to the authors’ knowledge, these variants have yet to be reported in one single specimen, and thus the potential clinical significance of these sole variants may be enhanced when possessed together.

Straight leg raise and femoral nerve traction tests are commonly performed orthopedic maneuvers done to ascertain the presence of a lumbar disc herniation [16,17]. Femoral nerve traction testing has a reported sensitivity of 50% and specificity of 100% for the diagnosis of midlumbar nerve root impingement, and appears to be insensitive and only 50% specific for lower lumbar nerve root impingement [18]. Straight leg raise testing has sensitivity and specificity characteristics of 16% and 31% respectively for midlumbar nerve root impingement. For the diagnosis of lower lumbar nerve root impingement, straight leg raise testing is 69% sensitive and 84% specific [18]. Reproduction of radicular leg pain in both sciatic and femoral nerve distributions with nerve traction testing is a common sign of lumbar disc herniations [16-18], and variations in both the course of the sciatic and femoral nerves as well as the surrounding musculature may affect the results of these nerve traction tests [2,4]. Recovery from radicular symptoms is often problematic and may be due to diagnostic problems in challenging cases. In a retrospective study conducted by Suri et al. in 2012, 81% of patients who sought conservative care for their leg pain associated with a lumbar disc herniation experienced resolution of symptoms in an average of 6 months. However, within 1 year post resolution, 25% had experienced a recurrence in their leg pain [19]. Patients who are refractory to care may warrant a reexamination, keeping in mind the many variations in anatomy, dermatomal patterns, and false positive/negatives of certain orthopedic tests [18,20-22]. A study of the distribution of dermatomal pain patterns by Murphy et al. showed 64.1% of the 169 lumbar spine pain patients presented with non-dermatomal pain distributions [23]. The sensitivity and specificity of lumbar spine dermatomal pain patterns associated with radiculopathies is too low to be useful in the identification of a specific nerve root level [23]. This is likely due to the communications between posterior collateral sensory ganglia and preganglionic neurons of different nerve root levels, creating variations in cutaneous sensations [22,24]. Several authors have concluded that variant femoral or sciatic nerve anatomy may produce a clinical picture analogous to that of a lumbar or lumbosacral radiculopathy [2,4,5,7,14]. Consideration of these anatomical variants, especially combined femoral and sciatic nerve variants, may prompt earlier or more focused diagnostic tests when a suspected lumbar spine disc herniation is refractory to care. One such test that may prove helpful to clinicians would be needle electromyography, as it can assist in the differentiation of radiculopathy and entrapment neuropathies [11].

Variants in lumbar and lumbosacral plexus anatomy should be considered when a symptomatic lumbar disc herniation is refractory to care. Recognition of these anatomical variants may lead to earlier intervention of physiologic testing, better treatment outcomes and improved patient satisfaction. Future studies examining the prevalence of these combined variants in the general population would be of interest to clinicians.

## Consent

Written informed consent was obtained from the deceased prior to the gift of body donation. All handling of anatomical specimens was in accordance with the institutions ethical policy for body donation for anatomical study and scientific purposes. A copy of the written consent is available for review by the Editor-in-Chief of this journal.

## Competing interests

The authors declare that they have no competing interests.

## Authors’ contributions

PB conceived of the case report, assisted in reviewing the literature and drafting the manuscript. FS provided anatomical artwork, assisted in reviewing the literature and drafting the manuscript. DE assisted in reviewing the literature, drafting the manuscript and provided critical review. All authors read and approved the final manuscript.
